# Knowledge and attitude towards strabismus among adult residents in Woreta town, North West Ethiopia: A community-based study

**DOI:** 10.1371/journal.pone.0278703

**Published:** 2022-12-02

**Authors:** Henok Biruk Alemayehu, Kalkidan Berhane Tsegaye, Fozia Seid Ali, Nebiyat Feleke Adimassu, Getasew Alemu Mersha

**Affiliations:** 1 Department of Ophthalmology and Optometry, College of Medicine and Health Sciences, Hawassa University, Hawassa, Ethiopia; 2 Department of Optometry, School Of Medicine, University Of Gondar, Comprehensive Specialized Hospital, Gondar, Ethiopia; 3 Hiwot Fana Specialized Hospital, Haramaya, Ethiopia; Cairo University Kasr Alainy Faculty of Medicine, EGYPT

## Abstract

**Background:**

Strabismus is a visual disorder where the eyes are misaligned and point in different directions. Untreated strabismus can lead to amblyopia, loss of binocular vision, and social stigma due to its appearance. Since it is assumed that knowledge is pertinent for early screening and prevention of strabismus, the main objective of this study was to assess knowledge and attitudes toward strabismus in Woreta town, Northwest Ethiopia. Providing data in this area is important for planning health policies.

**Methods:**

A community-based cross-sectional study was done in Woreta town from April–May 2020 with a sample size of 424. A systematic random sampling technique was employed to achieve the required sample size. A pre-tested self-administered questionnaire was used to collect the data. Data were entered using epi-data version 3.1, then processed and analyzed via SPSS version 20. Descriptive and analytical statistics were employed to summarize the data. A p-value of less than 0.05 was used to declare statistical significance.

**Result:**

A total of 401 individuals aged over 18 years participated, with a response rate of 94.5%. Of those who responded, 56.6% were males. Of all the participants, 36.9% were illiterate. The proportion of people with poor knowledge of strabismus was 45.1%. It was shown that 53.9% of the respondents had a favorable attitude. Older age, higher educational level, having a history of eye examination, and a having a family history of strabismus were significantly associated with good knowledge of strabismus. A higher educational level, older age, and hearing about strabismus were significantly associated with a favorable attitude toward strabismus.

**Conclusion and recommendation:**

The proportion of good knowledge and favorable attitude towards strabismus were lower than previously reported in Gondar City, Northwest Ethiopia. There is a need to provide health education and promotion campaigns on strabismus to the community: what strabismus is, its’ possible treatments and the need to bring children to the eye care center for early diagnosis and treatment.

## Introduction

Strabismus is a condition in which the eyes do not line up with one another. In other words, one eye is turned in a direction that is different from the other eye (ocular misalignment). This misalignment may be caused by abnormalities in binocular vision or by anomalies in neuromuscular control of ocular motility. Symptoms may include double vision, headaches, difficulty reading, eyestrain, and closing one eye when viewing faraway objects or when in bright light [1, 2].

Based on a pooled analysis done from studies around the globe, the prevalence of strabismus was 1.93% in the world, and it was 0.42% in Africa [3]. According to community-based studies in Ethiopia, the prevalence of strabismus was 1.53% to 5% among children in Butajira and Bahirdar Ethiopia, respectively [4, 5].

Untreated strabismus causes amblyopia, loss of binocular vision, cosmetic stigma, and psychosocial impact [6, 7]. Strabismus may create significant negative social prejudice and reduce a person’s chances of obtaining employment. Negative attitudes towards strabismus emerge at a young age, as early as 6 years [8]. It has been reported that strabismus adversely affects the parent-child relationship and a child’s psychological development [9–12].

Refractive error, anisometropia, cranial nerve palsy, assisted delivery (forceps or cesarean section), low birth weight and prematurity, neuro-developmental disorders, older maternal age at the time of childbirth, maternal smoking during pregnancy, and a family history of strabismus have all been associated with strabismus [12–14].

Treatment options typically include wearing glasses, patching the healthy eye in amblyopia to make the impaired eye work, and having surgery to fix the appearance of a squint [15]. However, poor parental knowledge, misconceptions, and misinformation adversely affect the age of presentation and management of strabismus [16].

A complete understanding of parents’ or guardians’ knowledge in this regard will help to design a strategy for early detection and management of strabismus. Unfortunately, this has received little attention from the scientific community, and very limited research has been done regarding this topic in general and the study area in particular. Few studies have been conducted in some areas of the country, most notably the Cheha district and Gondar in Northwest Ethiopia [17, 18]. Therefore, this study aimed to determine knowledge and attitude towards strabismus in adult residents at Wereta town, Northwest Ethiopia.

## Methods and materials

### Study design and period

A community-based cross-sectional study was conducted in Woreta town, which is located in Amhara Regional State, Northwest Ethiopia, about 614km from the capital Addis Ababa. The study was conducted from April–May 2020. The data obtained from Woreta city administration office showed that there were four kebeles with an estimated total population of 42,595 in 9229 households. Of these, 21,297 were females and 21,298 were males. Of the total population, 39,684 were adults, of which 19,921 were males and 19,763 were females. There were three private clinics and one governmental health center that provides health services, people with vision and eye problems including strabismus attended to the nearest eye care services at Bahir Dar and Gondar Referral eye care centers situated in Northwest Ethiopia.

### Sample size determination and sampling technique

The sample size was determined using a single population proportion formula by taking a 50% proportion of good knowledge, 95% confidence level, 5% margin of errors, and 10% non-response rate. Accordingly, the final computed sample size was 424. All four kebeles were included in the study. There were 42,595 people in total, with 39,684 adults and 9229 households. A sample fraction ’’k’’ was obtained by dividing the number of the household by the calculated sample size of 424. Systematic random sampling with proportional allocation was used to select the participating households with a sampling fraction (K) of 21 i.e. each household was approached in every 21 households included in the study. One individual was selected randomly from each household with more than one adult, using the lottery method to obtain a final sample size.

### Operational definition

The knowledge of strabismus was assessed using an 8-point scale questionnaire. There were 8 questions which has a total of 8 correct responses. Each correct response held a value of one the points were summed up to give a maximum response value of 8. The mean response value (i.e. 4) from the questionnaire was used as a cut of point to assess the knowledge of a respondent. Accordingly, the overall knowledge was categorized as good if respondents were able to score the mean score or above, and poor when respondents scored less than the mean [19].

The attitude was assessed by 7 questions using a 5-point Likert scale. The responses were summed up and a total score was obtained for each respondent. The mean score was calculated, and those who scored the mean or above the mean score had a favorable attitude, while scores below the mean were assessed as having an unfavorable attitude towards strabismus [19].

## Data collection procedure and quality control

Data was collected through a face-to-face interview with a pretested and semi-structured questionnaire which was translated from English to Amharic and back to English to maintain its consistency. The questionnaire had the following parts Part I: Contains 9 questions on socio-demographic characteristics including eye care service utilization and family history of strabismus, Part II: Contain10 in questions on awareness and knowledge of strabismus, and Part III: contains 7 attitude questions. Four optometrists participated in the data collection process. The data collectors randomly approached an individual for an interview in a selected household.

A pre-test was conducted in Gondar city northwest Ethiopia for validation by taking 5% of the sample size. Data quality was ensured through the training of data collectors and cross-checking of the filled questionnaire for completeness by the supervisors and principal investigator. The reliability of the questionnaires was checked, and the value of Cronbach’s alpha was 0.83.

### Data processing and analysis

The data was cleaned, coded, and entered using epi-data 3.1 and was exported to SPSS version 20 for processing and analysis. Analysis was done by the principal investigators using the same computer package. Proportions, frequency, ratios, and summary statistics such as mean, median, standard deviation, and range were used to summarize the data. Analytical statistics were performed to identify potential contributor variables. Crude and adjusted odds ratios were used to show the strength of the association between the dependent and independent variables. A 95% confidence interval and p-value of less than 0.05 was declared for statistical significance.

### Ethical considerations

Ethical clearance was obtained from the University of Gondar, the College of Medicine and Health Sciences, and the school of medicine ethical review committee. A letter of support from the Woreta town administration was obtained and a copy was given to the household head of the randomly selected households in the three kebeles. Oral informed consent was taken from each participant. The aim of the study was explained to the study subjects and their agreement was assured. All the information obtained was kept confidential by coding the data, and no personal identifiers were used.

## Results

### Socio-demographic characteristics

A total of 401 individuals aged >18 participated, with a response rate of 94.5%. Out of those who responded, 227(56.6%) were males, 55(13.7%) had no formal education, and 204 (50.9%) were single **(Table 1)**.

**Table 1:** Socio-demographic characteristics of adults living in Woreta town, Northwest Ethiopia, 2020 (n = 401).

Variables	Frequency	Percent (%)
**Age (year)**		
<24	96	23.9
24–32	113	28.3
33–43	94	23.4
>44	98	24.4
**Sex**		
Male	227	56.6
Female	174	43.4
**Marital Status**		
Single	204	50.9
Married	197	49.1
**Educational status**		
No formal education	55	13.7
Primary school	84	20.9
Secondary school	129	32.2
Higher education	133	33.2
**Occupational status**		
Unemployed	125	31.2
Employed	137	34.1
Private work	139	34.7
**Monthly income (ETB)**		
<2,000	136	33.9
2,000–3,200	67	16.7
3,201–5,000	104	25.9
>5,000	94	23.5

n = sample size ETB = Ethiopian Birr

### Knowledge towards strabismus

The results of our study indicated that 40.9% (95% CI: 36.2, 45.9%) had good knowledge of strabismus in this community.

Of 401 participants, 228(56.9%) of them had heard about strabismus, and a majority of them 101(25.2%) had obtained the information from their friends. Among those who reported knowing what strabismus is, 215 (53.6%) answered as ‘‘turning of the eye”.

Regarding the cause of strabismus, 279(69.6%) of participants incorrectly mentioned exposure to strong light as a cause of strabismus, while 224(55.9%) correctly mentioned trauma as a cause of strabismus. Among the respondents, 167 (41.6%) said that strabismus was treatable, while 159 (95%) of them stated the correct treatment options **(Table 2)**.

**Table 2:** Knowledge of strabismus among adult participants living in Woreta, Northwest Ethiopia, 2020 (n = 401).

Questions	Responses	
	Correct, n (%) Incorrect, n (%)	
What is strabismus	215 (53.6)	186 (46.4)
Cause of strabismus		
Communicable	356 (88.8)	45 (11.2)
Refractive error	92 (22.9)	309 (77.1)
Hereditary	309 (77.1)	92 (22.9)
Trauma	224 (55.9)	177 (44.1)
Strong light exposure	122 (30.4)	279 (69.6)
Is strabismus treatable	167 (41.6)	234(58.4)
How can it be treated	159 (95)	8 (5)
Overall knowledge of strabismus	Frequency, n (%)	
Good	164(59.1)	
Poor	237(40.9)	

n = sample size

### Attitude towards strabismus

**Fig 1:**
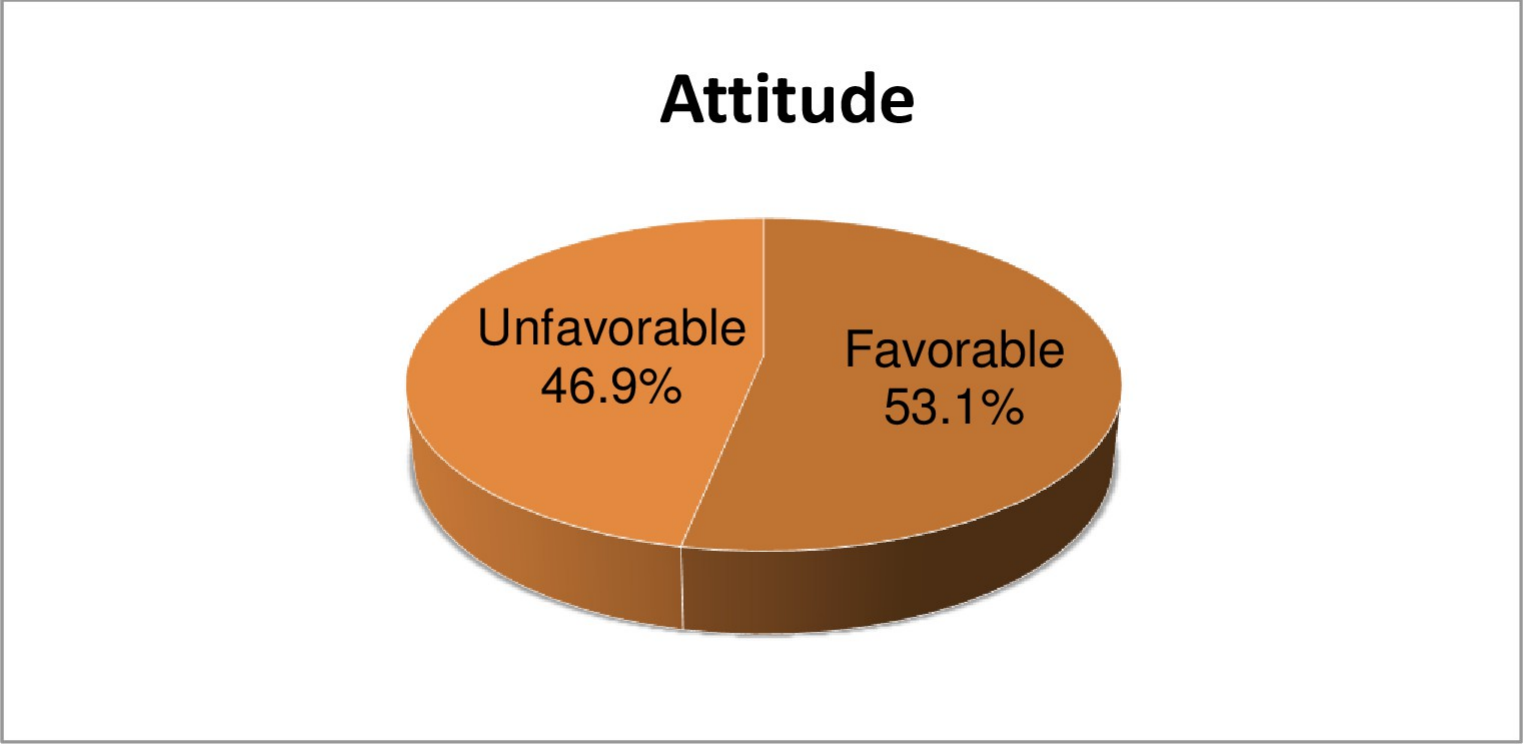
Pie chart showing attitude towards strabismus, among participants in Woreta town, Northwest Ethiopia, 2020 (n = 401).

Among the participants, 53.1% (95% CI: 48.4, 58.1%) had a favorable attitude towards strabismus **(Fig 1)**.

Regarding attitudes toward strabismus, 157 (39.2%) of respondents agreed to marry or allow relatives of a person with strabismus to be married to them. On the other hand, 224 (55.9%) of respondents strongly agreed that a child with strabismus can enter school and learn like other children; 283 (70.6%) of respondents strongly agreed that a person with strabismus can participate in social events, and 295 (73.6%) strongly agreed that a person with strabismus can participate in their special events **(Table 3)**.

**Table 3:** Attitude of respondents towards cases of strabismus concerning marriage, education and social life in Woreta town, Northwest Ethiopia, 2020 (n = 403).

Will Marry or allow the marriage of relatives to a person with strabismus	Frequency	Percent (%)
Strongly disagree	23	5.7
Disagree	81	20.2
Neutral	53	13.3
Agree	157	39.2
Strongly agree	87	21.6
**A child with strabismus Can enter school and learn**		
Strongly disagree	2	0.5
Disagree	15	3.7
Neutral	17	4.2
Agree	143	35.7
strongly agree	224	55.9
**A person with strabismus can participate in the social event**		
Strongly disagree	2	0.5
Disagree	1	0.2
Neutral	2	0.5
Agree	113	28.2
strongly agree	283	70.6
**Am happy if a person with strabismus participates at my special event**		
Strongly disagree	2	0.5
Disagree	2	0.5
Neutral	8	2.0
Agree	94	23.4
strongly agree	295	73.6

n = sample size

Approximately 46 (11.5%) of respondents agreed that strabismus is a divine curse, and 71 (17.7%) agreed that the cause of strabismus is caused by looking at strong sunlight or reflection from water, while 111(27.7%) of respondents strongly disagreed that looking sideways is the cause of strabismus **(Table 4)**.

**Table 4:** Attitude of respondents towards causes of strabismus concerning the cause of strabismus in Woreta town, Northwest Ethiopia, 2020 (n = 403).

Is a sign of bad luck or a curse from God	Frequency	Percent (%)
Strongly disagree	172	42.9
Disagree	149	37.2
Neutral	17	4.2
Agree	46	11.5
strongly agree	17	4.2
**Looking at strong sun light or reflection from water cause strabismus**		
Strongly disagree	129	32.2
Disagree	127	31.7
Neutral	26	6.4
Agree	71	17.7
strongly agree	48	12.0
**Do you believe looking sideways is a cause for strabismus**		
Strongly disagree	111	27.7
Disagree	111	27.7
Neutral	31	7.7
Agree	81	20.2
strongly agree	67	16.7

**n** = sample size

### Factors associated with good knowledge of strabismus

In multivariable logistic regression age, family history of strabismus, and history of eye examination were significantly associated with good knowledge of strabismus.

Participants aged >44 were 1.96 times (AOR = 1.96, 95% CI: 1.02, 3.78) more likely to have good knowledge about strabismus than participants of age < 24. Compared to those participants with no formal education, those with higher educational status were 3.88 times (AOR = 3.88, 95% CI: 1.71, 8.79) more likely to have good knowledge of strabismus. The likelihood of having good knowledge was 2.94 (AOR = 2.94, 95% CI: 1.26, 6.83) times higher for those with a family history of strabismus compared to those who didn’t have a family history of strabismus. Compared to those without a history of eye examination, participants with a history of eye examination had 4.48 times (AOR = 4.48, 95% CI: 2.64, 7.60) greater chances of having good knowledge **(Table 5)**.

**Table 5:** Factors associated with knowledge of strabismus among adult participants living in Woreta town, Northwest Ethiopia, 2020 (n = 401).

Variable	Knowledge Good poor		COR (95% CI)	COR (95% CI)	P value
**Sex**					
Male	92	135	1	1	
Female	72	102	1.04(0.69, 1.55)	1.07(0.65, 1.74)	0.793
**Educational status**					
No formal education	17	38	1	1	
Primary school	32	52	1.38(0.67, 2.83)	1.88(0.83, 4.24)	0.131
Secondary school	46	83	1.24(0.63, 2.44)	1.75(0.82, 3.78)	0.149
Higher education	69	64	2.41(1.24, 4.69)	3.88(1.71, 8.79)	**0.001**
**Age (year)**					
<24	34	65	1	1	
24–32	34	70	0.93(0.52, 1.66)	0.86(0.44, 1.69)	0.662
33–43	49	57	1.64(0.94, 2.89)	1.66(0.87, 3.19)	0.126
>44	47	45	1.99(1.12, 3.58)	1.96(1.02, 3.78)	**0.044**
**Family monthly income (ETB**)					
<2,000	59	77	1	1	
2,000–3,200	27	40	0.88(0.49, 1.59)	0.99(0.51, 1.93)	0.959
3,201–5,000	41	63	0.85(0.51, 1,43)	0.95(0.52, 1.73)	
>5,000	37	57	0.85(0.49, 1.45)	0.85(0.45, 1.58)	
**Marital status**					
Married	84	113	1	1	
Single	80	124	0.87(0.58, 1.29)	0.92(0.58, 1.47)	0.732
**Occupation status**					
Employed	54	71	1.31(0.80, 2.15)	1.39(0.73, 2.66)	0.591
Unemployed	59	78	1.30(0.80, 2.12)	1.27(0.69, 2.33)	
Private worker	51	88	1	1	
**Family history of strabismus**					
Yes	20	11	2.85(1.33, 6.13)	2.94(1.26, 6.83)	**0.012**
No	144	226	1	1	
**History of eye care visit**					
Yes	66	30	4.64(2.84, 7.62)	4.48(2.64, 7.60)	**0.0001**
No	98	207	1	1	

n = sample size ETB = Ethiopian Birr

### Factors associated with a favorable attitude toward strabismus

After running multivariable logistic regression, educational status, age, and marital status were significantly associated with a favorable attitude to strabismus.

It was found that participants with a higher educational level were 3.30 (AOR = 3.30, 95% CI: 1.53, 7.12) times more likely to have a favorable attitude towards strabismus than those with no formal education. Compared to participants who did not hear about strabismus, those who did were 1.72 times (AOR = 1.72, 95% CI: 1.12, 2.64) more likely to have a favorable attitude towards strabismus. Participants aged >44 were 3.45(AOR = 3.45, 95% CI: 1.80, 6.63) times more likely to have a favorable attitude to strabismus than those aged < 24 **(Table 6)**.

**Table 6:** Factors associated with attitude towards strabismus among adult participants living in Woreta town, Northwest Ethiopia, 2020 (n = 401).

Variable	Attitude		COR (95% CI)	AOR (95% CI)	P value
**Sex**					
Male	126	101	1	1	
Female	87	87	0.80(0.54, 1.19)	0.83(0.52, 1.31)	0.835
**Educational status**					
No formal education	20	35	1	1	
Primary school	40	44	1.59(0.79, 3.19)	1.97(0.91, 4.26)	0.60
Secondary school	73	56	2.28(1.19, 4.37)	2.77(1.34, 5.72)	**0.003**
Higher education	80	53	2.64(1.38, 5.56)	3.30(1.53, 7.12)	**0.001**
**Age (year)**					
<24	41	58	1	1	
24–32	49	55	1.26(0.72, 2.19)	1.23(0.66, 2.29)	0.501
33–43	62	44	1.99(1.14, 3.48)	1.70(0.92, 3.16)	0.089
>44	61	31	2.78(1.54, 5.02)	3.45(1.80, 6.63)	**0.0001**
**Family monthly income (ETB**)					0.713
<2,000	64	72	1	1	
2,000–3,200	40	27	1.66(0.92, 3.01)	1.43(0.81, 2.88)	
3,201–5,000	57	47	1.36(0.82, 2.28)	1.20(0.68, 2.71)	
>5,000	52	42	1.39(0.82, 2.36)	1.09(0.60, 1.96)	
**Marital status**					
Married	113	84	1	1	
Single	100	104	0.71(0.48, 1.06)	0.72(0.46, 1.12)	0.445
**Occupation status**					0.324
Employed	70	55	1.33(0.82, 2.16)	1.56(0.85, 2.86)	
Unemployed	75	62	1.26(0.79, 2.03)	1.14(0.65, 2.01)	
Private worker	68	71	1	1	
**Family history of strabismus**					
Yes	15	16	1	1	
No	198	172	0.81(0.39, 1.69)	0.73(0.32, 1.63)	0.445
**History of eye care visit**					
Yes	51	45	1.00(0.63, 1.58)	0.87(0.53, 1.45)	0.605
No	162	143	1	1	
**Heard any information about strabismus**					
Yes	108	65	1.94(1.30, 2.91)	1.72(1.12, 2.64)	**0.013**
No	105	123	1	1	

**n** = sample size **ETB** = Ethiopian Birr

## Discussion

Given the limited research done regarding knowledge and attitude towards strabismus in Ethiopia, adequate evidences were not found to comprehend on the level of knowledge of the community. Therefore, this study desired to provide some information about the knowledge and attitude towards strabismus of the community residing in some parts of North West Ethiopia.

In this study, the proportion of people with good knowledge of strabismus was 40.9% (95% CI: 36.2, 45.9%). The finding was lower compared to Saudi Arabia 50.6% [20] and Gondar, Ethiopia 52.3% [18]. This inconsistency might be attributed to the difference in the availability of eye care services and the educational level of participants. The presence of an eye care service in the vicinity would allow participants to have a frequent follow-up service that enhances their awareness and knowledge of the condition in the former studies [21]. In addition, the low awareness in the present study could be due to the source of information utilized, friends from whom the information might not be authenticated.

Regarding their detailed knowledge of strabismus, 56.6% of the participants had the correct definition of strabismus, congruent with a study done in the Western province of Saudi Arabia [22]. Most of the participants misunderstood that strong light exposure causes strabismus, similar to a study done in Cheha, Ethiopia [17]. Regarding the treatment of strabismus, 41.6% knew that strabismus is treatable. The figure was higher than the response from a study in the Cheha district 32% [17] and lower compared to a study done in Southwest Nigeria 50% [23]. This implies that it is crucial to enhance this part of knowledge, as it can cause delayed presentation and poor treatment outcomes in patients with strabismus.

In this study, 53.1% (95% CI: 48.4, 58.1%) had a favorable attitude towards strabismus. This was lower than the studies conducted in Gondar, Ethiopia (71.8%) and Saudi Arabia (70.4%) [20, 24]. Given that 91.9% of participants in the former study had heard about strabismus against 56.9% in the current study: participants who had heard about strabismus might have a lot of information and have a positive attitude toward strabismus.

In this study, older age and high educational status were found to be associated with good knowledge. This finding was supported by a study done in Hail, Saudi Arabia [25]. Participants with higher education status may have better learning opportunities about strabismus at different points in their lives. With advancing years, a person may acquire knowledge from a variety of sources, including society, relatives, and medical institutions [26].

A history of eye examination and a family history of strabismus was also found to be associated with good knowledge of strabismus. Participants who had a history of eye examination could obtain adequate information on strabismus that could help to boost their knowledge of this condition [27]. Besides, having a relative with strabismus in the family could help to prioritize knowledge of the condition [28].

Advanced age, higher educational level, and hearing any information about strabismus were associated with a favorable attitude toward strabismus. As people age, the level of social engagement will increase. This in turn made them open-minded toward strabismus. Being educated would influence a person’s attitude toward strabismus by making them ready to welcome new experiences and recognize many kinds of learning opportunities [29] that help them to build a favorable attitude toward strabismus.

Finally, as a limitation, the study questionnaire did not include questions concerning: awareness of the urgency for an eye exam in cases of strabismus, different treatment options like patching, glasses, surgery, and amblyopia that would enable comprehensively assessing the knowledge of eye turn.

## Conclusion

The proportion of good knowledge and favorable attitude towards strabismus were lower than previously reported in Gondar City, Northwest Ethiopia. There were also a lot of misconceptions in this community. There is a need to provide health education and promotion campaigns on strabismus to the community: what strabismus is, its’ possible treatments, and the need to bring children to the eye care center for early diagnosis and treatment. Besides, it is highly valuable to inform parents and guardians of the importance of early screening to rule out potential ocular conditions associated with the eye turn and treatment of amblyopia to achieve adequate vision with both eyes. It is equally important to involve health educators, health extension workers, and social coworkers in the health education and campaign process on various platforms including public gatherings.

## Supporting information

S1 File Strabismus questioner. (DOCX)

S1 Data Strabismus data. (SAV)
