# Supplementary material for: Knowledge and attitude towards strabismus among adult residents in Woreta town, North West Ethiopia: A community-based study
Source: PLoS One. 2022 Dec 2;17(12):e0278703. doi: 10.1371/journal.pone.0278703 (PMC9718407; doi:10.1371/journal.pone.0278703)
Supplement: S1 File — (DOCX) [file pone.0278703.s001.docx]

**Questionnaire on knowledge of diabetic retinopathy**

**General information**

Serial no.______________ Card No______Kebele_______

Data collection date________________________________

Name of data collector______________________________

Signature _______________________________________

| 01. | Age in years___________ | **Response** | **Remarks** |
| --- | --- | --- | --- |
| 02. | Sex | 1. Male 2. Female |  |
| 03. | Religion | 1. Orthodox 4. Catholic 2. Muslims 5. Other specify____ 3. Protestants |  |
| 04. | Marital status | 1. Single 3. Divorced 2. Married 4. Widowed 5.Separeted |  |
| 05. | Educational status | 1. Can’t read and write 2. Read and write 3. Primary school 4. Secondary school 5. College and above |  |
| 06. | Occupation | 1. Employed 5. House wife 2. Unemployed 6. Retired 3. Merchant 7.Other/s specify… 4. Farmer |  |
| 07. | Residence | 1. Urban 2. Rural |  |
| 08. | Monthly income in ETB | ___________ |  |

**Section I: Assessment of Socio-demographic and Economic variables**

**Section III: Questions related to diabetic follow up and eye check up**

| **S.No** | **Questions** | **Response** | **Remarks** |
| --- | --- | --- | --- |
| 01. | How long have you been with diabetes? | ______________Years |  |
| 02. | Is there anyone with diabetes in your family? | 1. Yes 2. No |  |
| 03. | How often do you visit the diabetes clinic? | 1. Every Month 2. Every 2 month 3. Every 3 Month 4. Every 6month 5. Every year 6. Others specify |  |
| 04. | Have you been examined your eye before? | 1. Yes 2. No |  |
| 05. | Have you been checked for your eye over the last one year | 1. Yes 2. No |  |

**Section III: Knowledge questions related to diabetic retinopathy**

| **S.No** | **Questions** | | **Response** | **Remarks** |
| --- | --- | --- | --- | --- |
| 01. | Diabetes can affects the eye | | 1. Yes 2. No 3. Don’t know | If yes go to Q2 |
| 02**.** | | Have you ever heard of diabetic retinopathy (damage to the retina/ nerve at the back of the eye due to diabetes)? | 1. Yes 2. No 3. Don’t know | If **yes,** go to Q3, unless go to the next participant. |
| 03**.** | | How did you first find out that diabetes can cause retinopathy? (Tick which ever applicable) | 1. Informed by physicians at   diabetes clinic   1. Informed by ophthalmologist 2. Got information from media 3. Got information from books 4. Got information from family 5. Got information from friends 6. Any other specify______ |  |
| 04**.** | | How many years after diagnosis of diabetes did you find out that diabetes can cause retinopathy? | 1. At the time of diagnosis 2. Any other time specify------ 3. Do not know |  |
| 05**.** | | Can diabetic retinopathy cause blindness? | 1. Yes 2. No 3. Do not know |  |
| 06**.** | | What are the factors that cause progression/worsening of diabetic retinopathy? (Tick which ever applicable) | 1. Poor control of diabetes  2. Hypertension  3. Nephropathy  4. Anemia  5. Do not know 6. Any other specify__ |  |
| 07. | | What is/are the treatment options available for diabetic retinopathy? (Tick which ever applicable) | 1. Spectacles 5. Do not know  2. Laser 6. Others specify  3. Injection in to the eye  4. Intraocular Surgery |  |
| 08**.** | | Can a person with diabetic retinopathy have normal vision? | 1. Yes 2. No 3. Do not know |  |
| 09**.** | | Should patients with diabetes have a periodic/regular dilated eye check up to look for diabetic retinopathy (examination of the back of the eye after instilling dilating eye drops to look for changes in the retina due to diabetes)? | 1. Yes 2. No 3. Do not know | If **No** or **Do not know** go to the next participants. |
| 10**.** | | How often should patients with diabetes who have no diabetic retinopathy have a dilated eye checkup? | 1. Once in 6 months 2. Once a year 3. Once in 2 years 4. Once in 5 years 5. Do not know 6. Any other (specify)_______ |  |

**Thank you!**
